# Supplementary material for: Analysis of PPARγ Signaling Activity in Psoriasis
Source: Int J Mol Sci. 2021 Aug 10;22(16):8603. doi: 10.3390/ijms22168603 (PMC8395241; doi:10.3390/ijms22168603)
Supplement: Supplementary file 1 [file ijms-22-08603-s001.zip › Supplemental materials_Analysis of PPARg signaling activity in psoriasis/Pathway models/Models images and html files/PPARG inhibits phenotypes assotiated with psoriasis/240874.html]

allergic contact dermatitis


# Disease allergic contact dermatitis

|  |  |
| --- | --- |
| URN | urn:agi-meshdis:Dermatitis%2c%20Allergic%20Contact |
| Total Entities | 0 |
| Connectivity | 1309 |
| Name | allergic contact dermatitis |

---

|  |  |
| --- | --- |
| Pathway | PPARG inhibits phenotypes assotiated with psoriasis |
|  | efects selected |

---

|  |  |
| --- | --- |
| MedScan ID | 9004672 |

---

|  |  |
| --- | --- |
| Alias | allergic skin condition |
|  | allergic skin lesions |
|  | allergic reactions in the skin |
|  | Eczematous Dermatitis, Allergic |
|  | skin allergic reactions |
|  | skin hypersensitivity |
|  | skin hypersensitivities |
|  | allergic skin disease |
|  | allergic reaction of the skin |
|  | allergic skin lesion |
|  | allergic diseases of the skin |
|  | cutaneous allergies |
|  | skin allergies |
|  | allergic skin inflammation |
|  | Dermatitis, Contact, Allergic |
|  | allergic dermatosis |
|  | Dermatitis, Allergic Contact |
|  | allergic skin reaction |
|  | Allergic Dermatitis |
|  | hypersensitivity in the skin |
|  | Allergic Contact Dermatitis |
|  | skin allergic reaction |
|  | allergic skin reactions |
|  | cutaneous allergy |
|  | Contact Dermatitis, Allergic |
|  | allergic skin diseases |
|  | Allergic Eczematous Dermatitis |
|  | allergic inflammations of the skin |
|  | allergic skin conditions |
|  | allergic dermatoses |
|  | allergy in the skin |
|  | skin allergy |
|  | Dermatitis, Allergic Eczematous |
|  | allergies in the skin |
|  | allergic inflammation of the skin |

---

|  |  |
| --- | --- |
| MeSH ID | D017449 |
|  | M0026474 |

---

|  |  |
| --- | --- |
| MeSH Heading | Dermatitis, Allergic Contact |

---
